# Interleukin-7 Facilitates HIV-1 Transmission to Cervico-Vaginal Tissue *ex vivo*

**DOI:** 10.1371/journal.ppat.1003148

**Published:** 2013-02-07

**Authors:** Andrea Introini, Christophe Vanpouille, Andrea Lisco, Jean-Charles Grivel, Leonid Margolis

**Affiliations:** 1 Eunice Kennedy-Shriver National Institute of Child Health and Human Development, National Institutes of Health, Bethesda, Maryland, United States of America; 2 Department of Biomedical Sciences and Technology, University of Milan, Milan, Italy; University of Pennsylvania School of Medicine, United States of America

## Abstract

The majority of HIV-1 infections in women occur through vaginal intercourse, in which virus-containing semen is deposited on the cervico-vaginal mucosa. Semen is more than a mere carrier of HIV-1, since it contains many biological factors, in particular cytokines, that may affect HIV-1 transmission. The concentration of interleukin (IL)-7, one of the most prominent cytokines in semen of healthy individuals, is further increased in semen of HIV-1-infected men. Here, we investigated the potential role of IL-7 in HIV-1 vaginal transmission in an *ex vivo* system of human cervico-vaginal tissue. We simulated an *in vivo* situation by depositing HIV-1 on cervico-vaginal tissue in combination with IL-7 at concentrations comparable with those measured in semen of HIV-1-infected individuals. We found that IL-7 significantly enhanced virus replication in *ex vivo* infected cervico-vaginal tissue. Similarly, we observed an enhancement of HIV-1 replication in lymphoid tissue explants. Analysis of T cells isolated from infected tissues showed that IL-7 reduced CD4^+^ T cell depletion preventing apoptosis, as shown by the decrease in the number of cells expressing the apoptotic marker APO2.7 and the increase in the expression of the anti-apoptotic protein B-cell lymphoma (Bcl)-2. Also, IL-7 increased the fraction of cycling CD4^+^ T cells, as evidenced by staining for the nuclear factor Ki-67. High levels of seminal IL-7 *in vivo* may be relevant to the survival of the founder pool of HIV-1-infected cells in the cervico-vaginal mucosa at the initial stage of infection, promoting local expansion and dissemination of HIV infection.

## Author Summary

Male-to-female HIV-1 transmission occurs predominantly through vaginal intercourse when the virus is transmitted with seminal fluid. The identification of the determinants of HIV-1 transmission to the female lower genital tract is of pivotal importance for understanding the basic mechanisms of HIV-1 infection. This understanding is necessary for the development of new strategies to prevent or contain this infection. Semen of HIV-1-infected individuals is highly enriched with IL-7, a crucial cytokine for the life cycle of CD4^+^ T cells, the primary target of HIV-1. Here, we utilized a system of human cervico-vaginal and lymphoid tissues *ex vivo* to study the effect of IL-7 on HIV-1 transmission and dissemination. Our results show that IL-7 at concentrations comparable to those found in semen of HIV-1-infected individuals enhanced HIV-1 replication by preventing the death and by stimulating the proliferation of CD4^+^ T cells. This allows sustained viral production by infected cells and provides new cellular targets for propagation of infection. The concentration of IL-7 in semen of HIV-1-infected men may be a key determinant of the efficiency of HIV-1 transmission to an uninfected female partner through vaginal intercourse.

## Introduction

HIV-1 male-to-female transmission occurs predominantly through vaginal intercourse and is mediated by semen [1]. Semen is more than a mere carrier of HIV-1, since it contains many biological factors that may facilitate or inhibit HIV-1 transmission [2], [3]. For example, semen harbors distinct amyloidogenic peptides that enhance HIV infection and likely contribute to HIV transmission [4], [5], and it contains cationic polypeptides that exhibit anti-HIV-1 activity [6]. Also, semen is rich in many cytokines [7] that may affect HIV-1 transmission [8]–[11].

We, and others, have previously reported that IL-7, one of the most prominent cytokines in semen of healthy fertile individuals [7], in seminal plasma can reach concentrations 100 times higher than in blood plasma [12], [13]. Moreover, in the course of HIV-1 infection the seminal plasma concentration of IL-7 is increased compared with that in uninfected individuals [12], [13].

IL-7 plays a central role in T cell development and homeostasis [14], and it is currently being evaluated as a treatment for severe lymphopenia in lymphoablative chemo- and radiotherapies and in the course of HIV-1 infection [15]. In particular, administration of IL-7 to HIV-1-infected individuals receiving antiretroviral therapy increased their blood T cell count [16], [17], although a transient blip of HIV-1 replication was observed in some patients. These and other reports [18], [19] focused on the effects of IL-7 on HIV-1-infected individuals, whereas, despite evidence of strikingly elevated IL-7 levels in seminal plasma, little is known on the effect of IL-7 on HIV-1 sexual transmission.

Here, we investigated the effect of IL-7 on HIV-1 infection of human cervico-vaginal and lymphoid tissue *ex vivo*. We found that IL-7 facilitates HIV-1 transmission and dissemination by preventing the apoptosis and promoting the proliferation of CD4^+^ T cells.

## Results

Below, we report on the effects of recombinant human IL-7 on human lymphoid (tonsillar) and cervico-vaginal tissues infected *ex vivo* with HIV-1. In particular, in these tissues we evaluated (i) HIV-1 replication, by assessing HIV-1 release in culture medium and the number of HIV-1-infected CD4^+^ T cells, (ii) CD4^+^ T cell death, by assessing cell depletion and evaluating the expression of the apoptotic marker APO2.7 and of the anti-apoptotic protein Bcl-2 in these cells, and (iii) CD4^+^ T cell proliferation, by measuring the expression of the nuclear protein Ki-67. Cervico-vaginal and lymphoid tissues infected *ex vivo* with HIV-1 were cultured in the presence of IL-7 at concentrations of 5 or 25 ng/mL, which are comparable with the concentrations of IL-7 found in semen of HIV-1-infected individuals [13].

### IL-7 enhances replication of laboratory strains and primary isolates of HIV-1 in human lymphoid and cervico-vaginal tissues *ex vivo*

**Figure 1:**
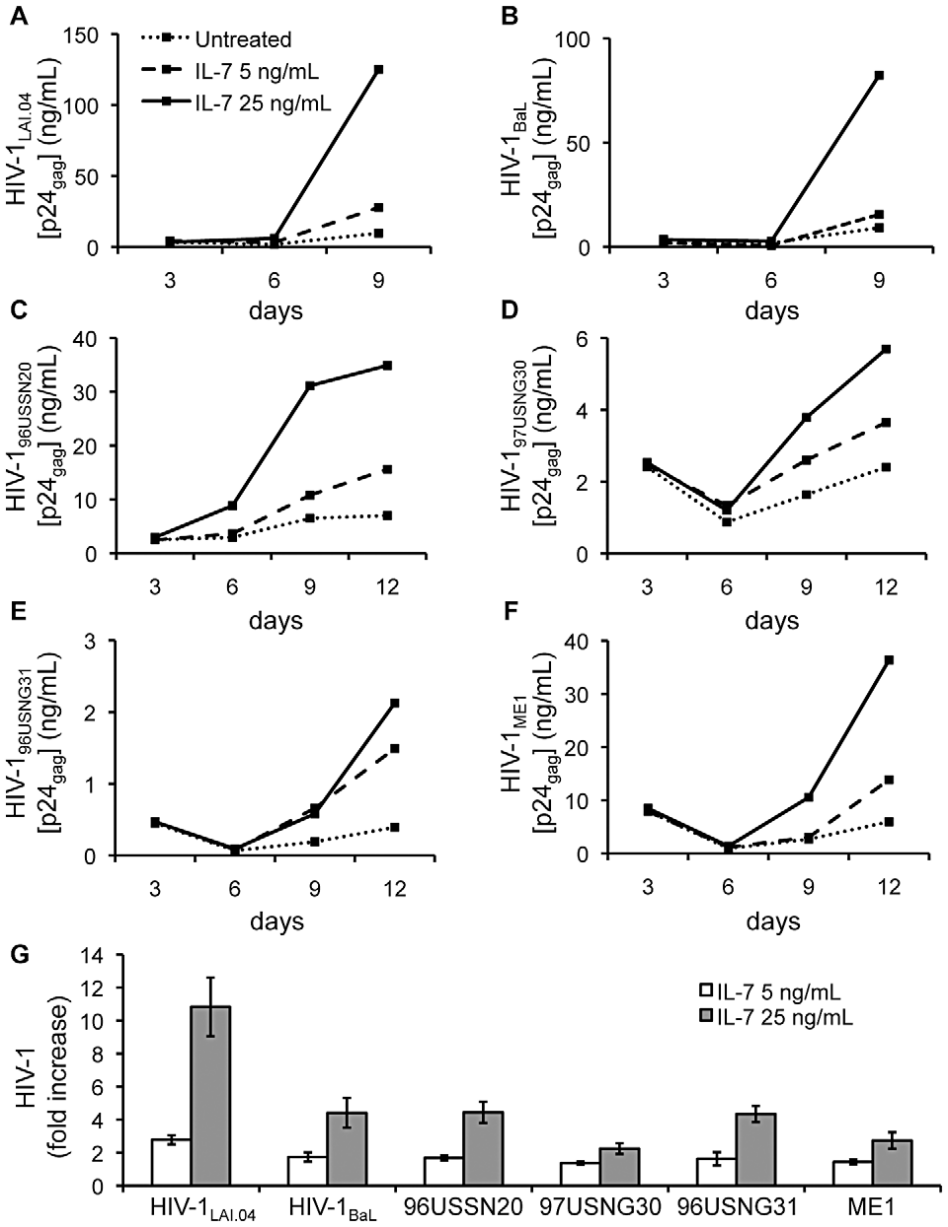
IL-7 enhances HIV-1 production in lymphoid tissue explants. Donor-matched lymphoid tissue blocks were inoculated with an X4 or R5 HIV-1 variant, HIV-1_LAI.04_ and HIV-1_BaL_ respectively, or with the primary isolates HIV-1_96USSN20_ (R5/X4 clade A), HIV-1_97USNG30_ (R5 clade C), HIV-1_96USNG31_ (R5/X4 clade C), and HIV-1_ME1_ (R5 clade B) and cultured for 9 or 12 days in the absence or presence of recombinant human IL-7 5 or 25 ng/mL. HIV-1 replication was monitored from measurements of HIV-1 p24_gag_ accumulated in culture media over 3-days periods. Presented are kinetics of the release of HIV-1 p24_gag_ in culture media of tissue blocks inoculated with HIV-1_LAI.04_ (**A**), HIV-1_BaL_ (**B**), and primary isolates (**C–F**) from representative donors. Each point represents pooled viral release from 27 tissue blocks over 3-days periods. (**G**) Presented are the average increases in the cumulative release of HIV-1 p24_gag_ in culture media of tissue blocks infected with different HIV-1 variants and treated with IL-7 5 ng/mL (*n* = 3–7) or 25 ng/mL (*n* = 7–13) compared with infected untreated donor-matched tissue blocks (means ± standard error of the mean (s.e.m.)).

IL-7 enhanced replication of HIV-1 isolates in lymphoid tissues infected *ex vivo* compared with donor-matched infected control tissues not exposed to IL-7. This enhancement was dose-dependent. Figure 1 (A, B) demonstrates the increase in replication of two prototypical CCR5- (R5) and CXCR4-utilizing (X4) HIV-1 strains, HIV-1_BaL_ and HIV-1_LAI.04_, in tissues treated with 5 and 25 ng/mL of IL-7. Figure 1 (C–F) demonstrates a similar phenomenon for the primary isolates HIV-1_96USSN20_ (clade A utilizing both CCR5 and CXCR4), HIV-1_97USNG30_ (clade C utilizing CCR5), HIV-1_96USNG31_ (clade C utilizing both CCR5 and CXCR4), and HIV-1_ME1_ (clade B utilizing CCR5). The absolute cumulative production of HIV-1 in controls varied in tissues from different donors and on average was 12.7±3.1 ng/mL for HIV-1_LAI.04_ and 7.3±0.9 ng/mL for HIV-1_BaL_. For primary isolates, HIV-1 production was on average 25.0±4.6 ng/mL, 3.9±0.4 ng/mL, 0.4±0.1 ng/mL, and 3.1±0.5 ng/mL for HIV-1_96USSN20_, HIV-1_97USNG30_, HIV-1_96USNG31_, and HIV-1_ME1_, respectively.

On average, IL-7 5 ng/mL significantly enhanced the production of HIV-1_LAI.04_ 2.8±0.3 fold (*n* = 3, *p*<0.01). The production of HIV-1_BaL_ was increased as well (1.7±0.3 fold), but did not reach statistical significance (*n* = 3, *p* = 0.094) (Figure 1G). IL-7 5 ng/mL enhanced the replication of primary isolates 1.7±0.1 fold, 1.4±0.1 fold, and 1.4±0.1 fold for HIV-1_96USSN20_, HIV-1_97USNG30_, and HIV-1_ME1_, respectively (*n* = 7, *p*<0.05). The production of HIV-1_96USNG31_ was also increased (1.6±0.4 fold), but did not reach statistical significance (*n* = 7, *p* = 0.153) (Figure 1G). There was a statistically significant increase in replication of all tested HIV-1 variants in tissues treated with IL-7 25 ng/mL: the increase was 10.8±1.8 fold for HIV-1_LAI.04_ (*n* = 13, *p*<0.001), and 4.4±0.9 fold for HIV-1_BaL_ (*n* = 9, *p*<0.001) (Figure 1G), and, for primary isolates, the increase was 4.4±0.6 fold, 2.2±0.3 fold, 4.3±0.5 fold, and 2.7±0.5 fold for HIV-1_96USSN20_, HIV-1_97USNG30_, HIV-1_96USNG31_, and HIV-1_ME1_, respectively (*n* = 7, *p*<0.005) (Figure 1G).

Also, a similar IL-7-mediated enhancement of HIV-1 replication was observed when viral inoculum was diluted 100 fold. In these experiments, the average cumulative production of HIV-1_LAI.04_ and HIV-1_BaL_ in untreated lymphoid tissues was 4.1±2.2 ng/mL and 2.7±1.3 ng/mL, respectively. In lymphoid tissues treated with IL-7 25 ng/mL HIV-1 replication increased 10.6±3.4 fold for HIV-1_LAI.04_ (*n* = 6, *p*<0.001), and 4.2±2.0 fold for HIV-1_BaL_ (*n* = 6, *p*<0.05). A similar increase was observed when HIV-1 viral stock was mixed with seminal fluid diluted 10 fold to diminish its *in vitro* toxicity [20]–[22], and then applied to lymphoid tissue blocks. IL-7 25 ng/mL increased replication of HIV-1_LAI.04_ and HIV-1_BaL_ 7.4±1.4 fold and 5.4±1.2 fold, respectively (*n* = 3, *p*<0.05).

**Figure 2:**
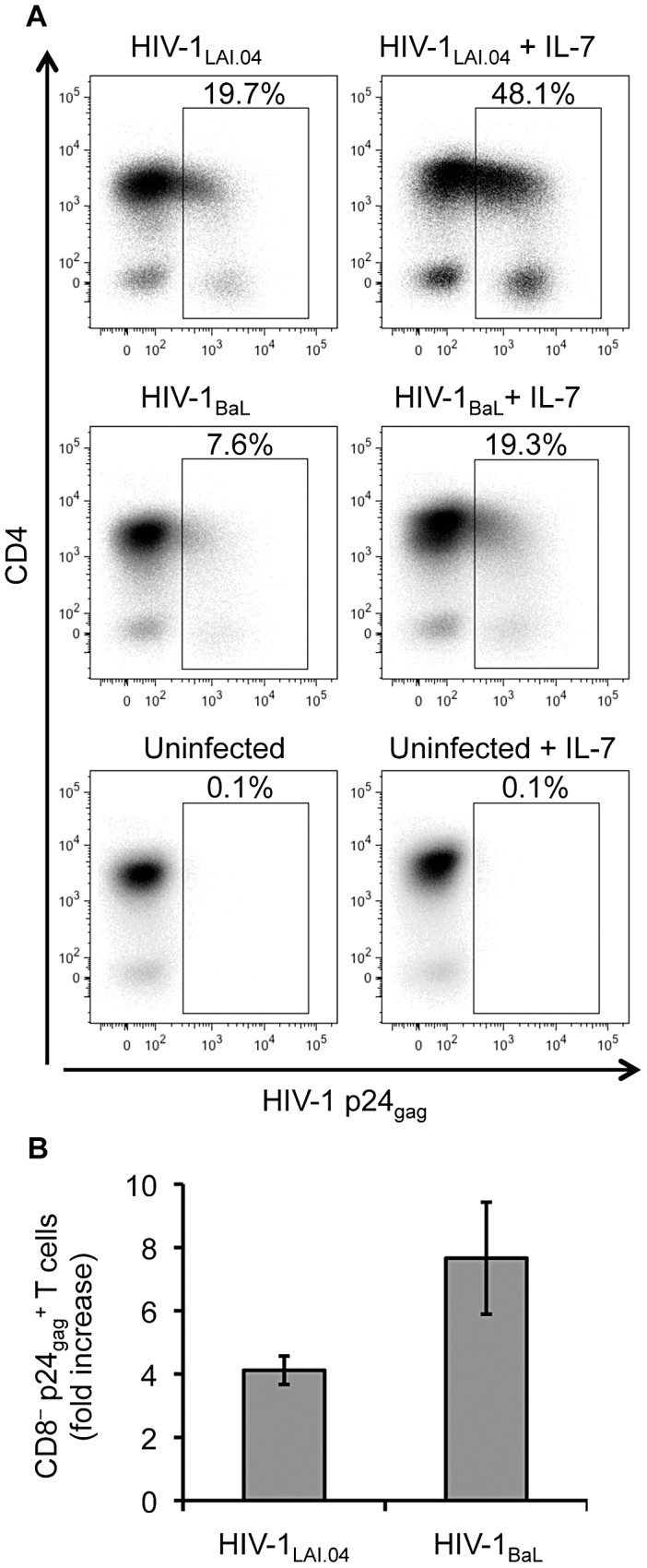
IL-7 increases the number of HIV-1-infected CD4^+^ T cells in lymphoid tissue explants. (**A**) Presented are dot plots for CD4^+^ T cells isolated from HIV-1-infected tissue blocks treated or not treated with IL-7 25 ng/mL from a representative donor on day 9 post infection. The amount of HIV-1-infected CD4^+^ (CD8^−^ p24_gag_^+^) T cells is expressed as percentage of CD3^+^ CD8^−^ cells. Upper panel: HIV-1_LAI.04_, middle panel: HIV-1_BaL_, lower panel: uninfected control. (**B**) Presented are the average increases in the numbers of HIV-1-infected CD4^+^ T cells isolated from tissue blocks infected with HIV-1_LAI.04_ (*n* = 8) or HIV-1_BaL_ (*n* = 6) and treated with IL-7 25 ng/mL compared with infected untreated donor-matched tissue blocks (means ± s.e.m.).

Consistent with the enhancement of HIV-1 replication, IL-7 increased the number of HIV-1-infected CD4^+^ T cells, as revealed by flow cytometry of tissue T cells stained intracellularly for HIV-1 p24_gag_ (Figure 2 A, B). As we previously described [23], to analyze CD4^+^ T cells we gated on CD8^−^ T cells to account for HIV-1-induced down-regulation of CD4. On average, IL-7 25 ng/mL increased the number of HIV-1-infected CD4^+^ (CD8^−^ p24_gag_^+^) T cells in HIV-1_LAI.04_- and HIV-1_BaL_-infected lymphoid tissues 4.1±0.4 fold and 7.7±1.8 fold, respectively, on day 9 post infection (*n* = 8 and 6, *p*<0.001) (Figure 2B).

**Figure 3:**
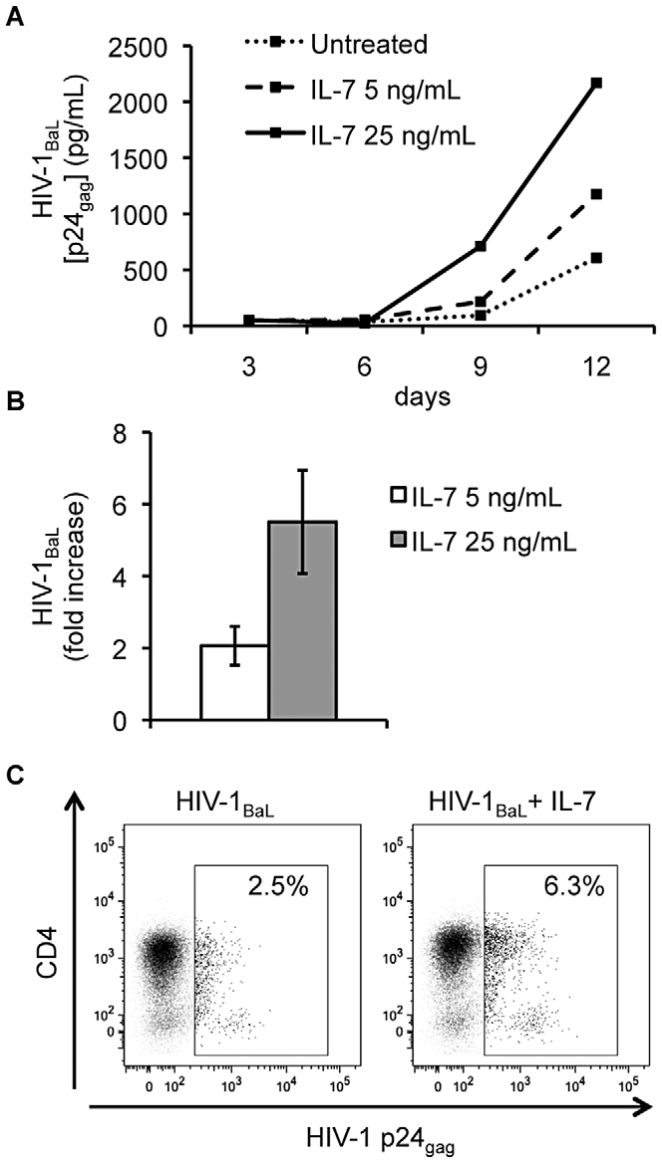
IL-7 increases HIV-1 production and the number of HIV-1-infected CD4^+^ T cells in cervico-vaginal tissue explants. Donor-matched cervico-vaginal tissue blocks were inoculated with HIV-1_BaL_ and cultured for 12 days in the absence or presence of IL-7 5 or 25 ng/mL. (**A**) Presented are kinetics of the release of HIV-1 p24_gag_ in culture media of tissue blocks inoculated with HIV-1_BaL_ from a representative donor. Each point represents pooled viral release from 24 tissue blocks over 3-days periods. (**B**) Presented are the average increases in the cumulative release of HIV-1 p24_gag_ in culture media of tissue blocks infected with HIV-1_BaL_ and treated with IL-7 5 or 25 ng/mL compared with infected untreated donor-matched tissue blocks (means ± s.e.m., *n* = 5). (**C**) Presented are dot plots for CD4^+^ T cells isolated from HIV-1-infected tissue blocks treated or not treated with IL-7 25 ng/mL from a representative donor on day 9 post infection. The amount of HIV-1-infected CD4^+^ (CD8^−^ p24_gag_^+^) T cells is expressed as percentage of total CD3^+^ CD8^−^ cells.

Also, IL-7 enhanced HIV-1_BaL_ replication in human cervico-vaginal tissues, which predominantly support productive infection of R5 rather than X4 HIV-1 variants [24]. This enhancement was first observed on day 9 post infection and became more prominent on day 12 (Figure 3A). On average, IL-7 25 ng/mL increased the production of HIV-1_BaL_ 5.5±1.4 fold (*n* = 5, *p*<0.01). IL-7 5 ng/mL increased HIV-1_BaL_ production 2.1±0.5 fold, but this increase did not reach statistical significance (*n* = 5, *p* = 0.129) (Figure 3B). The IL-7-mediated enhancement of HIV-1 replication was consistent for cervico-vaginal tissues from different donors and was observed even for a tissue in which HIV-1_BaL_ replication without IL-7 was as small as 75 pg/mL.

As with lymphoid tissues, IL-7 increased the number of HIV-1-infected CD4^+^ T cells in cervico-vaginal tissues infected with HIV-1_BaL_, as revealed by flow cytometry of tissue T cells stained intracellularly for HIV-1 p24_gag_ (Figure 3C). On average, IL-7 25 ng/mL increased the fractions of HIV-1-infected CD4^+^ (CD8^−^ p24_gag_^+^) T cells 3.0±0.9 fold on day 9 post infection (*n* = 5, *p*<0.05).

**Figure 4:**
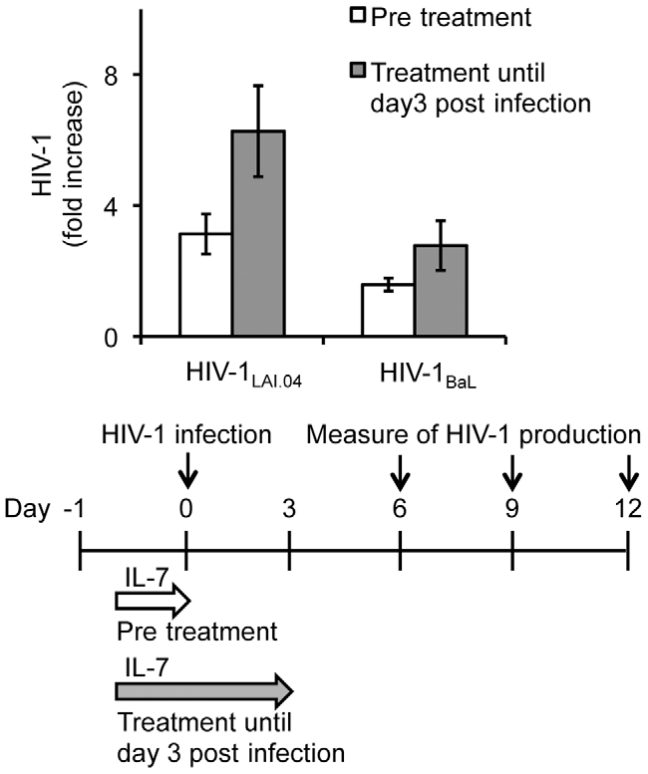
Exposure to IL-7 for a short time is sufficient to enhance HIV-1 production. Donor-matched lymphoid tissue blocks were treated with IL-7 overnight prior to infection with HIV-1 or until day 3 post infection, and subsequently cultured in the absence of IL-7. Presented are the average increases in the cumulative release of HIV-1 p24_gag_ in culture media of tissue blocks infected with HIV-1_LAI.04_ (*n* = 4) or HIV-1_BaL_ (*n* = 5) and treated with IL-7 25 ng/mL compared with infected untreated donor-matched tissue blocks (means ± s.e.m.).

In the above-described experiments, IL-7 was present throughout the entire culture period. Next, we investigated whether a short exposure of tissue to IL-7 was sufficient to enhance HIV-1 replication. In lymphoid tissues treated overnight with IL-7 25 ng/mL prior to infection with HIV-1_LAI.04_ and subsequently cultured in the absence of IL-7, HIV-1 replication was increased on average 3.1±0.6 fold (*n* = 4, *p*<0.05) (Figure 4). In lymphoid tissues pre-treated overnight with IL-7 and cultured in the presence of IL-7 until day 3 post infection, HIV-1_LAI.04_ replication was increased 6.3±1.4 fold (*n* = 4, *p*<0.01) (Figure 4). Similar experiments performed with HIV-1_BaL_ resulted in 1.6±0.2 fold increase (*n* = 5, *p*<0.05) in viral replication when tissues were pre treated with IL-7 and then cultured without IL-7, and 2.8±0.8 fold increase (*n* = 5, *p*<0.05) when tissues were pre treated with IL-7 and then cultured in the presence of IL-7 until day 3 post infection (Figure 4).

### IL-7 prevents the death and stimulates the proliferation of CD4^+^ T cells in HIV-1-infected tissues

We evaluated the effect of IL-7 on the size of the fraction of tissue CD4^+^ T cells by enumerating these cells in HIV-1-infected lymphoid tissues treated with IL-7 and in donor-matched infected untreated tissues with flow cytometry [25], [26]. HIV-1 depletes CD4^+^ T cells: on average, relative to uninfected donor-matched control tissues, HIV-1_LAI.04_ depleted 48.0±5.6% of CD4^+^ T cells after 9 days of infection. In donor-matched tissues treated with IL-7 25 ng/mL, the loss of CD4^+^ T cells was 3 times smaller, and HIV-1_LAI.04_ depleted 16.7±7.4% of CD4^+^ T cells (*n* = 7, *p*<0.0001). Thus, in HIV-1-infected lymphoid tissues treated with IL-7 the fraction of CD4^+^ T cells was bigger than in tissues cultured in the absence of IL-7. Next, we investigated whether this difference was due to IL-7 affecting the death or proliferation of CD4^+^ T cells.

**Figure 5:**
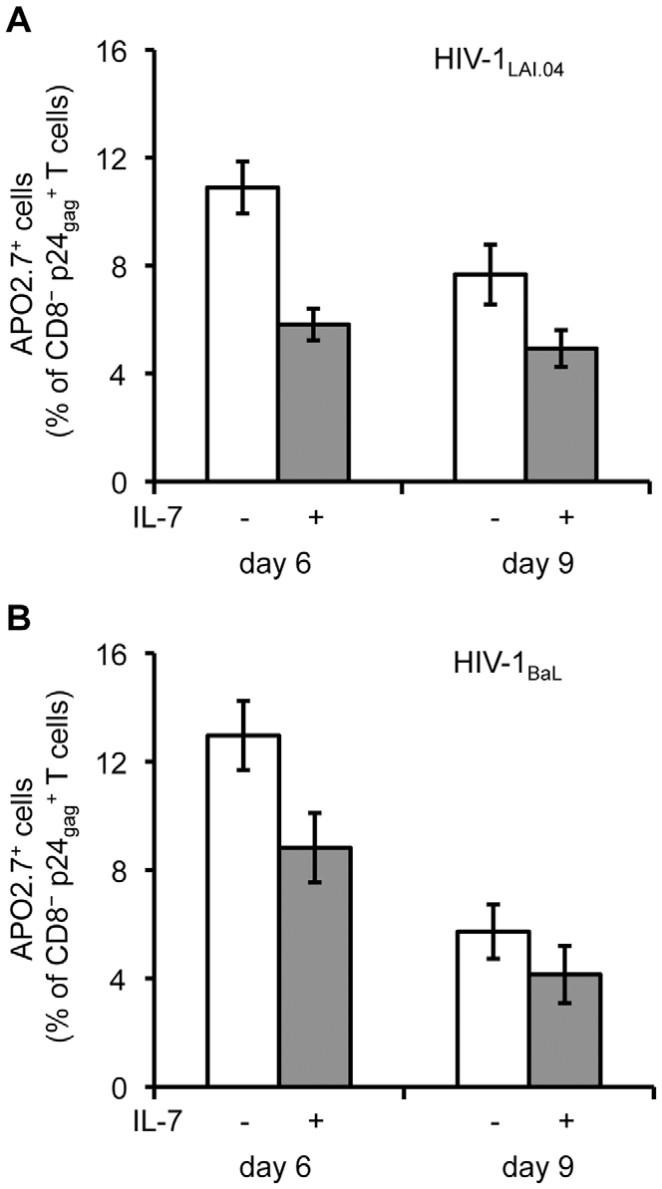
IL-7 decreases apoptosis of HIV-1-infected CD4^+^ T cells. Presented are the average fractions of HIV-1-infected CD4^+^ (CD8^−^ p24_gag_^+^) T cells expressing the apoptotic marker APO2.7 isolated from donor-matched lymphoid tissue blocks infected with HIV-1_LAI.04_ (*n* = 8) (**A**) or HIV-1_BaL_ (*n* = 5) (**B**) treated or not treated with IL-7 25 ng/mL (means ± s.e.m.). The amount of APO2.7^+^ HIV-1-infected CD4^+^ T cells is expressed as a percentage of total CD8^−^ p24_gag_^+^ T cells.

To investigate whether IL-7 treatment was associated with a lower incidence of CD4^+^ T cell apoptosis, we compared the expression of the apoptotic marker APO2.7 and the anti-apoptotic protein Bcl-2 in IL-7-treated and donor-matched untreated tissues infected with HIV-1_LAI.04_ or HIV-1_BaL_. On average, in lymphoid tissues treated with IL-7 25 ng/mL there was a decrease in the fraction of APO2.7-positive HIV-1-infected CD4^+^ (CD8^−^ p24_gag_^+^) T cells from 10.9±1.0% to 5.8±0.6% and from 7.7±1.1% to 5.0±0.7% of CD8^−^ p24_gag_^+^ T cells on day 6 and 9 post infection, respectively, for HIV-1_LAI.04_ (*n* = 8, *p*<0.001) (Figure 5A). Also, a decrease in the fractions of APO2.7-positive HIV-1-infected CD4^+^ T cells was observed in tissues infected with HIV-1_BaL_ from 13.0±1.3% to 8.8±1.3% and from 5.7±1.0% to 4.1±1.1% of CD8^−^ p24_gag_^+^ T cells on day 6 and 9 post infection, respectively (*n* = 6, *p*<0.05) (Figure 5B).

**Figure 6:**
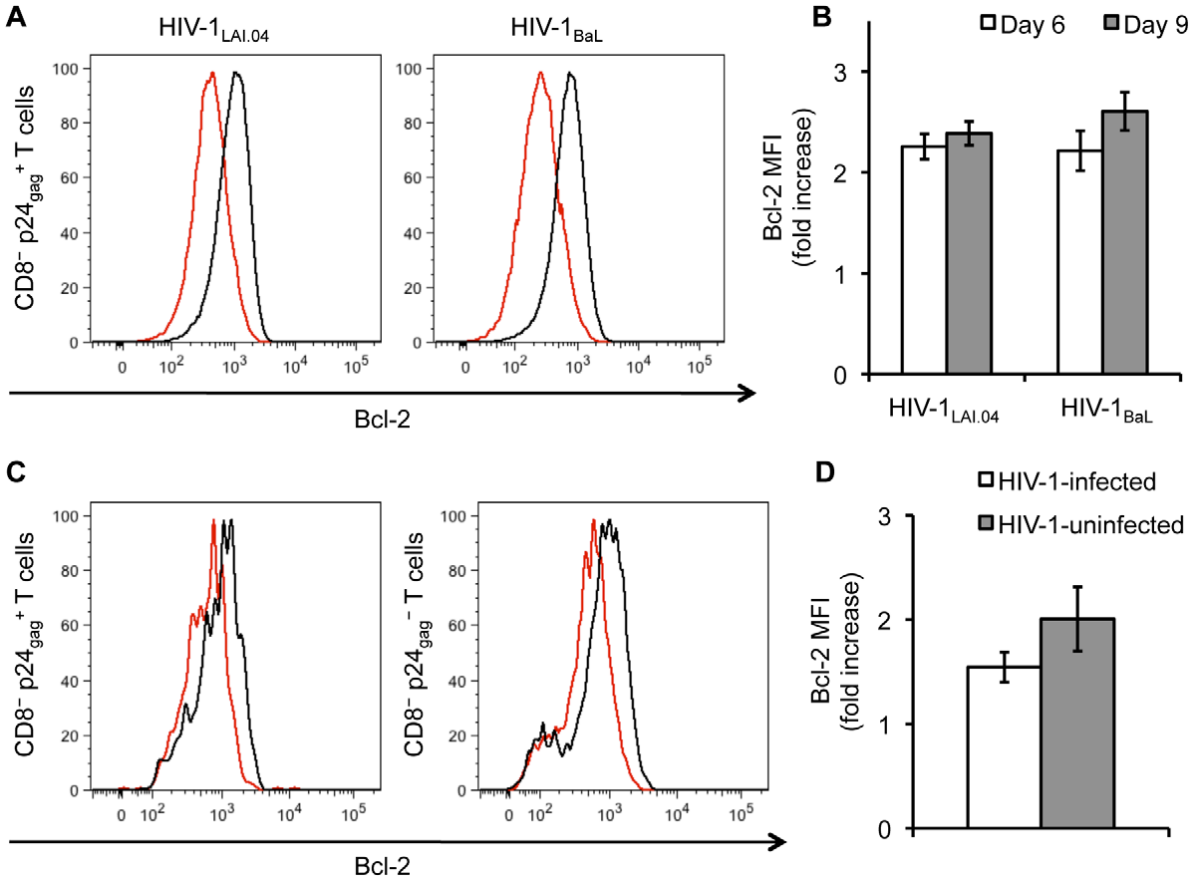
IL-7 up-regulates the expression of Bcl-2. (**A**) Presented are the expression levels of the anti-apoptotic protein Bcl-2 in HIV-1-infected CD4^+^ (CD8^−^ p24_gag_^+^) T cells isolated from lymphoid tissue blocks infected with HIV-1_LAI.04_ or HIV-1_BaL_ and treated with IL-7 25 ng/mL (black line) vs. infected untreated tissue blocks (red line) from a representative donor on day 9 post infection. The amount of Bcl-2 is expressed as median fluorescence intensity (MFI) value. (**B**) Presented are the average increases in Bcl-2 expression in HIV-1-infected CD4^+^ T cells isolated from lymphoid tissue blocks infected with HIV-1_LAI.04_ (*n* = 8) or HIV-1_BaL_ (*n* = 6) and treated with IL-7 25 ng/mL compared with infected untreated donor-matched tissue blocks (means ± s.e.m.). (**C**) Presented are the expression levels of Bcl-2 in HIV-1-infected and -uninfected (CD8^−^ p24_gag_^−^) CD4^+^ T cells isolated from cervico-vaginal tissue blocks infected with HIV-1_BaL_ and treated with IL-7 (black line) vs. untreated tissue blocks (red line) from a representative donor on day 9 post infection. (**D**) Presented are the average increases in Bcl-2 expression in HIV-1-infected and -uninfected CD4^+^ T cells isolated from cervico-vaginal tissue blocks infected with HIV-1_BaL_ and treated with IL-7 25 ng/mL compared with infected untreated donor-matched tissue blocks (means ± s.e.m., *n* = 5).

Consistent with the down-regulation of the apoptotic marker APO2.7, IL-7 increased the expression of the anti-apoptotic protein Bcl-2 in HIV-1-infected CD4^+^ T cells. Since Bcl-2 is highly expressed by all mature T cells [27], we compared the levels of its expression by measuring the median fluorescence intensity (MFI) (Figure 6A). In HIV-1_LAI.04_-infected lymphoid tissues, IL-7 25 ng/mL increased Bcl-2 expression in HIV-1-infected CD4^+^ T cells on average 2.3±0.1 fold and 2.4±0.1 fold on day 6 and 9 post infection, respectively (*n* = 8, *p*<0.0001) (Figure 6B). For HIV-1_BaL_-infected lymphoid tissues, IL-7 increased Bcl-2 expression in HIV-1-infected CD4^+^ T cells 2.2±0.2 fold and 2.6±0.2 fold on day 6 and 9 post infection, respectively (*n* = 6, *p*<0.001) (Figure 6B). A similar effect was observed in cervico-vaginal tissues infected with HIV-1_BaL_. IL-7 25 ng/mL increased Bcl-2 expression in HIV-1-infected CD4^+^ T cells on average 1.5±0.1 fold on day 9 post infection, compared with donor-matched infected untreated tissues (*n* = 4, *p*<0.05) (Figure 6 C, D).

In both lymphoid and cervico-vaginal tissues, the anti-apoptotic effect of IL-7 was not limited to HIV-1-infected CD4^+^ T cells but was observed in uninfected CD4^+^ T cells as well. In HIV-1-infected lymphoid tissues treated with IL-7 25 ng/mL Bcl-2 expression in uninfected CD4^+^ (CD8^−^ p24_gag_^−^) T cells was increased on average 2.5±0.2 fold and 2.7±0.1 fold on day 6 and 9 post infection, respectively, for HIV-1_LAI.04_ (*n* = 8, *p*<0.0001), and 2.6±0.1 fold and 3.0±0.1 fold on day 6 and 9 post infection, respectively, for HIV-1_BaL_ (*n* = 6, *p*<0.0001). In HIV-1_BaL_-infected cervico-vaginal tissues IL-7 25 ng/mL increased Bcl-2 expression in uninfected CD4^+^ T cells on average 2.0±0.3 fold on day 9 post infection (*n* = 4, *p*<0.05) (Figure 6 C, D). The increase in Bcl-2 expression was accompanied by the decrease in APO2.7 expression. On average, in HIV-1-infected lymphoid tissues treated with IL-7 25 ng/mL the fraction of uninfected CD4^+^ T cells expressing APO2.7 decreased from 10.8±0.9% to 6.6±0.6% and from 11.0±1.2% to 8.3±0.8% of CD8^−^ p24_gag_^−^ T cells on day 6 and 9 post infection, respectively, for HIV-1_LAI.04_ (*n* = 8, *p*<0.01), and from 8.0±0.8% to 5.5±0.7% and from 4.6±0.8% to 3.6±0.8% of CD8^−^ p24_gag_^−^ T cells on day 6 and 9 post infection, respectively, for HIV-1_BaL_ (*n* = 6, *p*<0.05).

**Figure 7:**
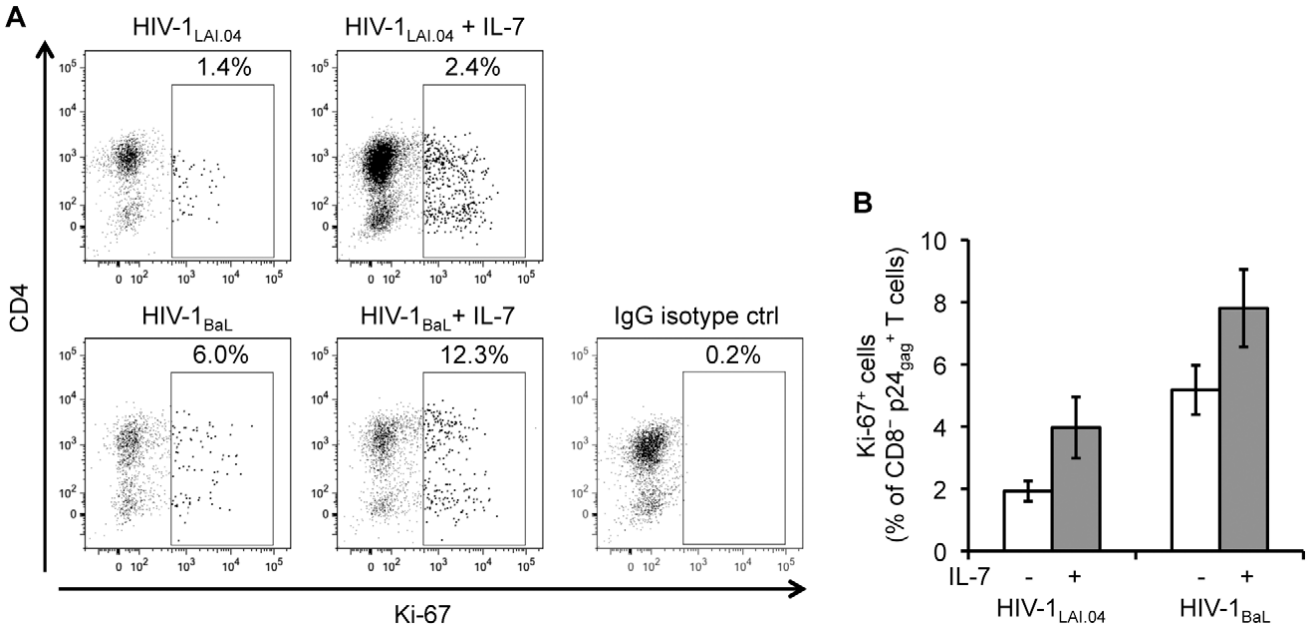
IL-7 increases the proliferation of HIV-1-infected CD4^+^ T cells. (**A**) Presented are dot plots for HIV-1-infected CD4^+^ (CD8^−^ p24_gag_^+^) T cells isolated from lymphoid tissue blocks infected with HIV-1_LAI.04_ or HIV-1_BaL_ treated or not treated with IL-7 25 ng/mL from a representative donor on day 9 post infection. The amount of Ki-67^+^ HIV-1-infected CD4^+^ T cells is expressed as percentage of total CD8^−^ p24_gag_^+^ T cells. (**B**) Presented are the average fractions of HIV-1-infected CD4^+^ T cells expressing Ki-67 isolated from donor-matched lymphoid tissue blocks infected with HIV-1_LAI.04_ or HIV-1_BaL_ treated or not treated with IL-7 25 ng/mL (means ± s.e.m., *n* = 6).

To investigate whether the increased number of CD4^+^ T cells in tissues treated with IL-7 compared with donor-matched untreated tissues was also associated with cell proliferation, we enumerated cycling cells by flow cytometry using the nuclear protein Ki-67 as marker of proliferation. In HIV-1-infected lymphoid tissues IL-7 25 ng/mL increased the fraction of Ki-67-positive HIV-1-infected CD4^+^ T cells on average 2.3±0.6 fold, from 1.9±0.3% to 4.0±1.0% of CD8^−^ p24_gag_^+^ T cells for HIV-1_LAI.04_ (*n* = 6, *p*<0.05), and 1.5±0.2 fold, from 5.2±0.8% to 7.8±1.2% of CD8^−^ p24_gag_^+^ T cells for HIV-1_BaL_ on day 9 post infection (*n* = 6, *p*<0.05) (Figure 7 A, B). In these tissues, IL-7 also increased the fraction of Ki-67-positive uninfected CD4^+^ T cells on average 7.1±1.2 fold, from 0.7±0.1% to 5.2±1.5% of CD8^−^ p24_gag_^−^ T cells for HIV-1_LAI.04_ (*n* = 6, *p*<0.0001), and 6.7±1.2 fold, from 0.8±0.1% to 5.9±1.6% of CD8^−^ p24_gag_^−^ T cells for HIV-1_BaL_ on day 9 post infection (*n* = 6, *p*<0.001).

## Discussion

Male-to-female HIV-1 transmission through vaginal intercourse is a complex process that depends on the ability of HIV-1 to infect its target cells in the mucosa of the female lower genital tract. The immunological milieu of the female genital mucosa plays a critical role during the early stages of HIV-1 transmission [28], and semen actively affects this milieu [2], [3]. In particular, semen contains many cytokines and we, among others, have found that the seminal cytokine network is deeply modified by HIV infection [12], [13]. Although semen from HIV-1-infected individuals is enriched in IL-7 [12], [13], little is known about the role of this cytokine in HIV sexual transmission.

IL-7 is a member of the common gamma-chain cytokine family comprising IL-2, IL-4, IL-15, and IL-21 [29]. IL-7 has crucial and non-redundant functions in modulating T cell development and peripheral naïve and memory T cell homeostasis [14], [30], [31]. The main source of IL-7 in semen is believed to be the prostate, where IL-7 is important for maintaining T cells residing in prostate-associated lymphoid tissue [32]. Specifically, in the current work, we simulated the *in vivo* situation by depositing HIV-1 on *ex vivo* cervico-vaginal tissue together with IL-7 at concentrations comparable to those measured in semen of HIV-1-infected individuals [13]. Epithelial cells isolated from the female genital tract and immortalized, namely endocervical epithelial cells, have been described as releasing IL-7, whereas ectocervical and vaginal epithelial cell lines do not produce IL-7 [33]. In culture media of cervico-vaginal tissue blocks *ex vivo*, we did not detect IL-7 over 12 days of culture, which allowed us to study the role of this cytokine in HIV-1 transmission *ex vivo*.

In our study we also used lymphoid (tonsillar) tissue *ex vivo* to assess viral dissemination and HIV-1-induced CD4^+^ T cell depletion. Reticular stromal cells isolated from human tonsils have been reported to produce IL-7, which localizes at the cell surface to be presented to naïve T cells [34]. However, there are no data on the absolute amount of IL-7 released by isolated stromal cells *in vitro*. In our experiments, integral tonsillar tissue blocks did not secrete detectable amount of this cytokine in culture medium, suggesting that produced IL-7 remains on the surface of stromal cells or is readily taken up by neighboring T cells.

We found that IL-7 significantly enhanced replication of the R5 variant HIV-1_BaL_ in cervico-vaginal explants from all the donors tested in this study, including one tissue sample in which, according to our criteria (see Materials and Methods), there was no replication in the untreated tissue. Similarly, we observed an enhancement of HIV-1 replication in lymphoid tissue infected with both R5 and X4 HIV-1 variants, since unlike cervico-vaginal tissue *ex vivo*, lymphoid tissue supports replication of HIV-1 of both phenotypes. This enhancement became detectable on day 6 post infection and seems to be independent of the absolute level of viral replication, as it was also observed in tissues infected with a viral inoculum diluted 100 fold. The enhancement of HIV-1 replication by IL-7 was not restricted to laboratory strains of this virus but was also observed for primary isolates. Moreover, this enhancement was observed for HIV-1 isolates of all three tested clades. Among these isolates were mono CCR5-tropic and dual CXCR4/CCR5-tropic HIV-1 variants. Viruses of the former tropism are thought to be preferentially transmitted and to dominate early stages of infection, while the latter are characteristic of later stages of HIV-1 disease [35], [36]. Thus, IL-7 seems to be an enhancer of replication of different HIV-1 variants replicating at different levels in different human tissues.

In general, our results are in agreement with previously reported IL-7-mediated enhancement of HIV-1 replication in primary mature thymocytes [27] and in PBMCs isolated from chronically infected patients, upon *in vitro* stimulation [37]. Also, it has been reported that IL-7 is able to induce HIV-1 permissiveness in quiescent T cells [38] and can reactivate latent HIV-1 in resting CD4^+^ T cells isolated from infected individuals [39], [40].

In our experiments, although the magnitude of HIV-1 enhancement was proportional to the length of exposure to IL-7, we found that IL-7 does not need to be present during the entire culture period to up-regulate HIV-1 infection. Moreover, when tissues were exposed to IL-7 prior to HIV-1 infection only, subsequent HIV-1 replication was enhanced.

Similarly, IL-7 enhanced HIV-1 replication when infection was performed on the background of human seminal fluid. It is known that semen collected with currently available protocols (ejaculated, coagulated, liquefied, frozen, and thawed) is toxic *in vitro* [20]–[22]. Therefore, for these experiments we used lymphoid tissue, where HIV-1 replication is more robust than in cervico-vaginal tissue. Although experiments on HIV-1 transmission to cervico-vaginal tissue blocks in the context of freshly ejaculated semen from HIV-1-infected men would be more informative, such experiments are difficult to perform due to logistic and ethical obstacles. Nevertheless, our data, being extrapolated to male-to-female *in vivo* HIV-1 transmission, suggest that a high concentration of IL-7 in semen may render the female lower genital tract mucosa more susceptible to HIV-1 acquisition.

What are the mechanisms of IL-7-mediated facilitation of HIV-1 infection?

Our data indicate that IL-7 prevents tissue CD4^+^ T cell depletion by suppressing their apoptosis. Consistent with the IL-7-mediated prolongation of the life of CD4^+^ T cells [14], we observed a general and persistent increase in the expression of the anti-apoptotic protein Bcl-2 in CD4^+^ T cells, both infected and uninfected. Another evidence of suppression of apoptosis by IL-7 was the decrease in the number of CD4^+^ T cells expressing the apoptotic marker APO2.7. Thus, IL-7 not only prolonged the life of cells that produce virus, thus allowing a continuous release of HIV-1, but it also prevented death of uninfected CD4^+^ T cells, thus providing HIV-1 with more potential targets.

The reduction of apoptosis by IL-7 observed in *ex vivo* tissues is in general agreement with the reduction of spontaneous apoptosis in T cells obtained from HIV-1-infected individuals, upon IL-7 treatment *in vitro* [41], and with the recent data on IL-7 treatment of rhesus macaques in the acute phase of simian immunodeficiency virus (SIV) infection in the absence of antiretroviral therapy [42]. In the latter study, IL-7 administration resulted in reduced depletion of circulating CD4^+^ T cells and in sustained increase in the expression of Bcl-2, associated with a transient increase in SIV replication.

However, the number of HIV-1-producing cells was increased in IL-7-treated tissues not only because of suppression of apoptosis but, as our data on Ki-67 expression indicate, also because IL-7 induced the proliferation of CD4^+^ T cells, both infected and uninfected. The IL-7-mediated promotion of cell proliferation has been reported for cultures of isolated cells [17], [41], [43]–[45] and is the basis of the current clinical trial aimed to increase T cell count in HIV-1 infected individuals [16], [17].

In our experiments, the two described mechanisms through which IL-7 facilitates HIV-1 infection, the prevention of apoptosis and the promotion of CD4^+^ T cells proliferation, resulting in the increase in the number of cells producing virus and in the duration of production, seem to be sufficient to explain the observed phenomenon of up-regulation of HIV-1 infection by IL-7. Additional mechanisms may contribute to the effect of IL-7 on HIV-1 tissue infection. These mechanisms may include up-regulation of co-stimulatory molecules on CD4^+^ T cells [37], direct induction of HIV-1 LTR transcription [40], increased expression of the coreceptor CXCR4 on CD4^+^ T cells [45]–[47], and induction of permissiveness of quiescent T cells for HIV infection [38].

Although the system of cervico-vaginal and lymphoid tissue *ex vivo* seems to adequately simulate the main hallmarks of HIV-1 infection, such as the size of the pool of HIV-1 infected CD4^+^ T cells, the depletion of CD4^+^ T cells by HIV-1, etc., it has limitations. In particular, in *ex vivo* tissues there is no recruitment of immune cells, which may play an important role in establishing HIV-1 infection. Cervico-vaginal explants are not polarized, and both IL-7 and HIV have immediate access to the inner mucosal cells, although *in vivo* HIV-1 is also thought to have access to these cells through lesions in the epithelial layer of the female genital mucosa. Finally, in our model we used cell-free HIV-1, while according to some reports cell-associated HIV-1 also may be transmitted *in vivo* [48].

Although our data indicate that IL-7 promotes HIV-1 transmission to cervico-vaginal tissue and its dissemination in lymphoid tissue, there is no apparent contradiction of these data with the beneficial effect of IL-7 for HIV-1-infected individuals [19]. Indeed, Levy et al. reported that subcutaneous administration of IL-7 leads to a dose-dependent CD4^+^ T cell increase in HIV-1-infected subjects receiving antiretroviral therapy, although transient low HIV viremia was seen in 6 of 26 patients [16]. In spite of this beneficial effect, in those individuals who shed virus in semen an elevated level of seminal IL-7 may increase the probability of HIV-1 transmission to their sexual partners.

In summary, although the effect of IL-7 on HIV replication in isolated cells as well as the effects of IL-7 on individuals already infected with HIV-1 have been reported, our study is the first to address the role of IL-7 in HIV-1 male-to-female transmission. Our results demonstrate that exposure of cervico-vaginal and lymphoid tissues *ex vivo* to IL-7 at concentrations comparable with those found in semen of HIV-1-infected individuals facilitates HIV-1 infection. The IL-7-mediated enhancement of HIV-1 production is associated with the proliferation and prevention of apoptosis of infected CD4^+^ T cells as well as of new potential target cells for HIV-1. These effects may be important for HIV-1 transmission because, unlike lymphoid tissue, the cervix contains a relatively low number of CD4^+^ T cells [24], which *in vivo* are the primary targets for HIV-1 infection [28]. Therefore, the increase in the number and the prolongation of the lifespan of HIV-1-infected cells, together with uninfected cells, will result in expanding the founder pool of infected cells, thus enhancing the risk of HIV-1 acquisition.

IL-7 seems to belong to a group of soluble factors [4], [5] that facilitate HIV-1 transmission. The concentration of these facilitating factors in semen of HIV-1-infected men may be a key determinant of the efficiency of HIV-1 transmission to uninfected partners through vaginal intercourse.

## Materials and Methods

### Tissue culture *ex vivo*

Tonsillar tissues from routine tonsillectomies were obtained from the Children's Hospital (Washington, DC). Cervico-vaginal tissues were obtained from routine hysterectomy through the National Disease Research Interchange (NDRI, Philadelphia, PA). All tissues were anonymous pathological samples obtained according to an Institutional Review Board-approved protocol.

Tonsillar tissues were dissected into approximately 8-mm^3^ blocks and placed on collagen sponge gels in culture medium at the air-liquid interface in a 6-well plate (9 blocks/well in 3 mL of RPMI1640 supplemented with 20% fetal bovine serum (FBS, Gemini Bioproducts, West Sacramento, CA). For each experimental condition, from 18 to 27 blocks were used, depending on the experiment. Tissue blocks were infected by application of 6.3 µL of viral stock, undiluted or diluted 100 fold in RPMI1640, on top of each block [49]. In some experiments the infection was performed by application of viral stock mixed 1∶1 with 20% seminal fluid (European sperm bank USA, Seattle, Washington).

The mucosal epithelium and the underlying stroma of both the ectocervix and the endocervix were separated from muscular tissue and dissected into approximately 8-mm^3^ blocks. For each experimental condition 24 cervical blocks were transferred into two 1.5-mL conical tubes (12 blocks per tube), each containing 0.5 mL of viral stock HIV-1_BaL_. After 2 hours of incubation at 37°C, tissue blocks were gently washed three times with 4 mL of phosphate-buffered saline (PBS) and placed on top of a collagen sponge gel in a 12-well plate (8 blocks/well in 1 mL of RPMI1640 supplemented with 20% FBS).

Tonsillar and cervico-vaginal tissue blocks were cultured for 6, 9, or 12 days (depending on the goal of the experiment) in the presence or absence of 5 or 25 ng/mL of recombinant human IL-7 (Peprotech, Rocky Hill, NJ) with a change of medium every 3 days.

### Viruses

HIV-1_BaL_ and HIV-1_LAI.04_ viral preparations were obtained from the Virology Quality Assurance Laboratory at Rush University (Chicago, IL). Viral stocks were obtained from the medium of peripheral blood mononuclear cell cultures inoculated with either HIV-1_BaL_ or HIV-1_LAI.04_, originally received from the NIH AIDS Reagent Program. HIV-1 p24_gag_ concentrations were 49±3 ng/mL and 53±3 ng/mL for HIV-1_BaL_ and HIV-1_LAI.04_ stock, respectively.

Viral stocks of HIV-1 clinical isolates were obtained through the NIH AIDS reagent program: deposited in the program by Drs D. Ellenberger, P. Sullivan, and R.B. Lal (HIV-1_96USSN20_, HIV-1_97USNG30_, and HIV-1_96USNG31_) [50], and by Dr. Phalguni Gupta (HIV-1_ME1_) [51]. HIV-1 p24_gag_ concentrations were 182±12 ng/mL, 275±23 ng/mL, 97±6 ng/mL, and 914±49 ng/mL for HIV-1_96USSN20_, HIV-1_97USNG30_, HIV-1_96USNG31_, and HIV-1_ME1_, respectively.

### Flow cytometry

Tonsillar and cervico-vaginal tissue blocks were digested for 30 and 45 minutes respectively, with Liberase low Dispase concentration (Roche, Indianapolis, IN) at final concentration of 8 µg/mL in 1 mL of RPMI1640 containing DNase I (Roche) at final concentration of 100 µg/mL. Single-cell suspensions were washed in staining buffer (PBS supplemented with 2% normal mouse serum; Gemini Bioproducts). To characterize tissue lymphocytes, cell suspensions were stained with different combinations of the monoclonal antibodies anti-CD3 Qdot 655, anti-CD4 Qdot 605, and anti-CD8 Pacific Blue (Invitrogen, Carlsbad, CA). After surface staining, cells were permeabilized with the Fix & Perm Kit (Invitrogen) and stained with the monoclonal antibodies anti-HIV-1 p24_gag_ fluorescein isothiocyanate (Beckman Coulter, Fullerton, CA), anti-Bcl-2 phycoerythrine (PE) (BD Bioscience, San Jose, CA), and anti-APO2.7 PE-Cy5 (Beckman Coulter). Cells were stained with the monoclonal antibody anti-Ki-67 PE (BD Bioscience) upon permeabilization with the FOXP3 Fix/Perm Buffer Set (Biolegend, San Diego, CA).

Data were acquired on an LSRII flow cytometer (BD Biosciences) equipped with 355-, 405-, 488-, 532-, and 638-nm laser lines using DIVA 6.1.2 software (BD Biosciences), and analyzed with FlowJo version 9.4.10 (Tree Star, Ashland, OR). We identified and excluded dead cells from the analysis using the LIVE/DEAD fixable Blue Dead Cell Stain kit (Invitrogen) and identified lymphocytes according to their light-scattering properties. We quantified cell depletion by using the AccuCheck counting beads (Invitrogen) according to the manufacturer's instructions.

### Dynamic immunofluorescent cytometric bead assay for HIV-1 p24_gag_ quantification

We evaluated productive HIV-1 infection from measurements of HIV-1 p24_gag_ antigen in medium of tonsillar and cervico-vaginal tissue cultures, using a dynamic immunofluorescent cytometric bead assay. The assay was performed as described previously [52].

### Statistical analysis

We conducted statistical analysis using the software GraphPad Prism (Version 4.0c). To account for inter-donor variability, we calculated the ratios between IL-7-treated and IL-7-untreated donor-matched conditions of: the values of HIV-1 p24_gag_ concentrations in culture media, the numbers and percentages of CD8^−^ p24_gag_^+^ T cells, the percentages of APO2.7^+^ T cells, Ki-67^+^ T cells, and the values of Bcl-2 MFI. For CD4^+^ T cell depletion, we calculated the difference in the percentages of CD3^+^ CD8^−^ cells between IL-7-treated and IL-7-untreated donor-matched conditions. Ratio and difference values were first log(n) transformed to be normally distributed, as verified by the Shapiro-Wilk normality test, and One-sample t tests were used to test for a nonzero mean. All tests were two-tailed, and a *p*-value of <0.05 defined statistical significance.

### Accession numbers

The UniProtKB (http://www.uniprot.org/) accession numbers for the proteins discussed in this paper are BCL2_HUMAN (P10415), IL-7_HUMAN (P13232), KI67_HUMAN (P46013), and HIV-1 p24_gag_ (Q7ZBG8).
